# Eight-Chop Technique in Phacoemulsification Using Iris Hooks for Patients with Cataracts and Small Pupils

**DOI:** 10.3390/jcm13237298

**Published:** 2024-11-30

**Authors:** Tsuyoshi Sato

**Affiliations:** Department of Ophthalmology, Sato Eye Clinic, Nemoto 3-3, Matsudo-shi 271-0077, Chiba-ken, Japan; overlord1944@m8.gyao.ne.jp

**Keywords:** cataract surgery, eight-chop technique, iris hooks, phacoemulsification, small pupil

## Abstract

**Objectives**: This study investigated the efficacy and safety of performing phacoemulsification using the eight-chop technique with iris hooks in patients with small pupils. **Methods:** The iris hooks and control groups each included 65 eyes. Cataract surgeries were performed using the eight-chop technique. The operative time, phaco time, aspiration time, cumulative dissipated energy, and volume of fluid used were measured. Best-corrected visual acuity, corneal endothelial cell density (CECD), and intraocular pressure (IOP) were measured preoperatively and postoperatively. **Results:** In total, 130 eyes of 107 patients (mean age, 75.9 ± 7.1 years; 58 men, 72 women) with cataracts were evaluated. The mean operative time, phaco time, aspiration time, cumulative dissipated energy, and volume of fluid used were 10.6 min, 20.7 s, 101.1 s, 7.8, and 38.0 mL, respectively, in the iris hooks group and 4.6 min, 16.2 s, 72.1 s, 7.0, and 28.9 mL, respectively, in the control group. The decrease in CECD at 19 weeks postoperatively was 2.1% and 1.2% for the iris hooks and control groups, respectively. In both groups, IOP decreased significantly (all *p* < 0.01) at 7 and 19 weeks postoperatively. No intraoperative complications were found in either group. **Conclusions:** The eight-chop technique using iris hooks resulted in a small postoperative reduction in CECD and excellent values for intraoperative outcome measures. In addition, those cataract surgeries were very short, efficient, and safe, without complications. The eight-chop technique using iris hooks could provide an ideal solution for patients with small pupils.

## 1. Introduction

Cataract surgery is one of the most common surgeries in the world [1,2]. However, phacoemulsification performed through a small pupil represents a significant challenge for surgeons, and the procedure is generally regarded as being associated with a higher incidence of complications [2,3]. Small pupils are observed in approximately 4.4–11% of all cataract operations [2,4,5], with a reported complication frequency of 4.7–9.0% [5,6]. Thus, the use of any established method of intraoperative widening of the pupil by the surgeon is considered practical. The essential elements of a successful procedure are the adequate retraction of the iris when required and the safe navigation of the pupil. A number of techniques have been developed to widen a small pupil during phacoemulsification. The use of iris hooks offers the advantage of maintaining a stable pupil size throughout the surgical procedure [7].

The evolution of phacoemulsification can be traced back to the single-handed engraving technique, which gave way to the divide-and-conquer technique, first introduced by Gimbel [8]. This was followed by the phaco-chop technique, as described by Nagahara [9], and the prechop technique, as presented by Akahoshi [10]. The prechop and eight-chop techniques manually divide the nucleus under an ophthalmic viscosurgical device prior to phacoemulsification [10,11]. In comparison to alternative techniques, the total ultrasound energy is markedly diminished, and the aspiration time and the volume of fluid used are considerably reduced [10,11]. The eight-chop technique is distinguished by the separation of the lens nucleus into eight segments instead of four as in the prechop technique [11]. Furthermore, this technique facilitates safe maneuvering during phacoemulsification, even within a small pupil, because the divided lens nucleus is small and holding an ultrasound handpiece with both hands allows you to operate the ultrasound tip more precisely.

To date, no study has been conducted utilizing the eight-chop technique for patients with small pupils. Moreover, no studies have examined the intraoperative parameters in patients with small pupils; thus, the surgical details of these difficult cases are unclear.

The present study was designed to evaluate a series of intraoperative outcome measures, postoperative best-corrected visual acuity (BCVA), corneal endothelial cell density (CECD), and intraocular pressure (IOP) changes for phacoemulsification procedures performed using the eight-chop technique with iris hooks in patients with small pupils (≥6 mm) and to compare the results obtained with those of patients with pupils ≥6 mm and results reported previously [2,3,4,5,6,7].

## 2. Materials and Methods

### 2.1. Study Population

This study included patients with cataract who underwent phacoemulsification and posterior chamber intraocular lens implantation at our clinic between March 2015 and February 2022. Patients with a preoperative pupil diameter of ≥6 mm were considered for the control group, whereas patients with a pupil diameter of <6 mm were considered for the iris hooks group [6]. Patients were excluded if they had corneal disease or opacity, uveitis, or a history of trauma or surgery.

### 2.2. Preoperative Assessment

Preoperatively, all patients received a comprehensive ophthalmic examination, including a slit-lamp and retinal assessment, and their BCVA and IOP were documented. CECD was quantified using a noncontact specular microscope (EM-3000; Topcon Corporation, Tokyo, Japan). The degree of firmness of the nucleus was evaluated according to the Emery classification system [12]. The same surgeon, proficient in the eight-chop technique, conducted a phacoemulsification procedure utilizing the phacoemulsification unit (Centurion^®^; Alcon Laboratories, Inc., Irvine, CA, USA).

### 2.3. New Surgical Instruments

**Figure 1:**
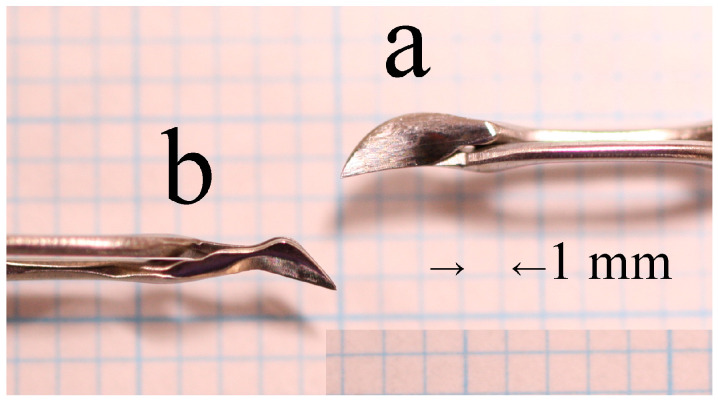
Eight-choppers: (**a**) the Eight-chopper I (SP-8193; ASICO, Parsippany, NJ, USA); (**b**) the Eight-chopper II (SP-8402; ASICO).

A new generation of surgical instruments (Figure 1) has been developed to facilitate the implementation of the eight-chop technique [11]. The research team devised a design for eight choppers and requested that a manufacturing company undertake the production process. The Eight-chopper I (SP-8193; ASICO, Parsippany, NJ, USA) features a reduced diameter tip in comparison to a conventional prechopper. With a length and width of 3.2 mm and 1.4 mm, respectively, and a more pronounced leading edge, the Eight-chopper I was employed for the grade II group. The Eight-chopper II (SP-8402; ASICO) is characterized by a smaller angular tip (2.5 mm long and 0.8 mm wide) that can be effectively inserted vertically into the lens nucleus. This particular device was utilized for the grade III group.

### 2.4. Surgical Technique

**Figure 2:**
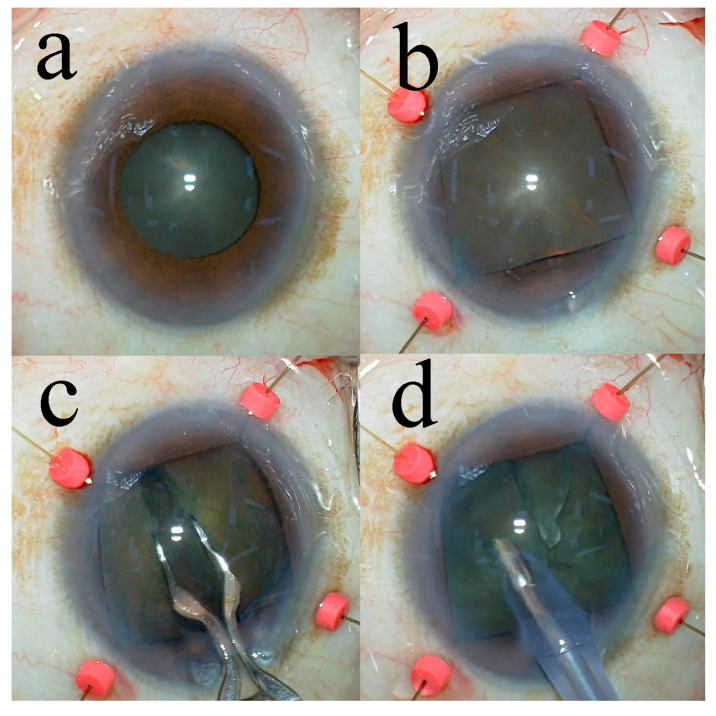
The phacoemulsification procedure. (**a**) The pupil diameter is 5.6 mm before installation of the iris hooks. (**b**) Four iris hooks are installed. (**c**) The eight-chop technique is performed by installing the iris hooks. (**d**) Phacoemulsification is performed after dividing the lens nucleus into eight sections.

In all surgical procedures, a temporal clear corneal incision was created using a 3.0-mm steel keratome. Following the injection of sodium hyaluronate into the anterior chamber, a continuous curvilinear capsulorhexis with a diameter of 6.2–6.5 mm was created using capsule forceps. For the iris hooks group, iris hooks (Synergetics, Inc., O’Fallon, MO, USA) were used to retract the iris through corneal stab incisions from four directions (Figure 2). The grade III group was treated with the soft-shell technique [13]. A 27-gauge cannula was utilized for hydrodissection; however, hydrodelineation was not conducted. The lens nucleus was divided into eight segments employing the Eight-chopper I or II for eyes categorized as grade II or III, respectively. An ophthalmic viscosurgical device was placed in the anterior chamber, and the eight-chopper was then inserted into the center of the lens nucleus, which was completely divided. After the nucleus was completely divided into two pieces, the lens nucleus was rotated 90 degrees. The eight-chopper was inserted into the center of the heminucleus, which was completely divided. The lens nucleus was rotated 180 degrees to complete four divisions of the lens nucleus, then rotated 45 degrees and divided four times to complete eight divisions of the lens nucleus. At the depth of the iris plane, the eight segments were phacoemulsified and aspirated. The capsular bag was evacuated with the irrigation and aspiration tip, facilitating the removal of cortical materials. The ophthalmic viscosurgical device was introduced and a foldable intraocular lens (Acrysof^®^ MN60AC; Alcon Laboratories, Inc., Fort Worth, TX, USA) with polymethylmethacrylate haptics was placed into the capsular bag using an injector system. The ophthalmic viscosurgical device was then removed by aspirating. The phacoemulsification unit was used in all the surgeries. It had a maximum ultrasound power of 80%, a rate of 32 mL/min and a 1.1 mm tip. If necessary, the wound was closed with corneal stromal hydration. After the surgery, the anterior chamber was exchanged with a balanced salt solution containing moxifloxacin (0.5 mg/mL).

### 2.5. Outcome Measures and Data Collection

The intraoperative outcome measures were the operative time in minutes, phaco time in seconds, aspiration time in seconds, cumulative energy dissipated, volume of fluid used in mL, and intraoperative complication rate. From the beginning of the corneal incision to the end of the aspiration of the ophthalmic viscosurgical device, the operative time was counted. From the creation of the first corneal stab incision to the placement of the fourth hook, hooking time was counted. All the surgeries were recorded, and the pupil diameters were analyzed based on the surgical videos according to the method by Vasavada et al. [14]. Follow-up was performed on postoperative days 1 and 2 and at 1, 3, 7, and 19 weeks. BCVA, IOP, and CECD (cells/mm^2^) were the postoperative outcome measures. BCVA, CECD, and IOP were measured 7 and 19 weeks postoperatively. The iris hooks and control groups were divided into subgroups for analysis based on preoperative IOP: those with IOP above 15 mmHg and those with IOP below 15 mmHg according to the method of Poley et al. [15].

### 2.6. Statistical Analysis

Unpaired *t*-tests were used for statistical analysis to compare results between the iris hooks and control groups. Preoperative BCVA, CECD, and IOP were compared with each postoperative time point using paired *t*-tests. The level of statistical significance was set at *p* < 0.05. The chi-square test was used to determine any differences in sex, diabetes mellitus, and intraoperative floppy iris syndrome incidence between the iris hooks and control groups.

## 3. Results

This study included 130 eyes of 107 patients with cataract who underwent phacoemulsification and posterior chamber IOL implantation. The characteristics of the patients and the intraoperative parameters are presented in Table 1. Between the iris hooks and control groups, no significant differences in mean age were found. No significant differences were found in the incidence of diabetes mellitus; however, significant differences were found in the incidence of intraoperative floppy iris syndrome and gender between the iris hooks and control groups. Pupil size was found to be significantly different between the iris hooks and control groups before and after surgery. Operative times, phaco times, irrigation times, and volume of fluid used differed significantly between iris hooks and control groups. However, between the iris hooks and control groups, no significant differences in cumulative energy dissipated were found.

Table 2 lists the pre- and postoperative changes in the BCVA measured. Between the iris hooks and control groups, no significant differences in BCVA were found preoperatively and 19 weeks postoperatively. However, a significant difference in BCVA was found between the iris hooks and control groups at 7 weeks postoperatively. The BCVA between preoperatively and 7 weeks postoperatively, between 7 weeks and 19 weeks postoperatively, and between preoperatively and 19 weeks postoperatively were significantly different in the iris hooks group. Moreover, the BCVAs between preoperatively and 7 weeks postoperatively and preoperatively and 19 weeks postoperatively in the control group differed significantly; however, in the control group, there were no significant differences in BCVA between 7 and 19 weeks postoperatively.

Table 3 lists the pre- and postoperative changes in the CECD measurements. Preoperative CECDs were not significantly different between the iris hooks and control groups. However, significant differences in CECD were found between the iris hooks and control groups at 7 and 19 weeks postoperatively. There was a significant difference in CECD between preoperatively and 7 weeks postoperatively and preoperatively and 19 weeks postoperatively in the iris hooks group. However, in the iris hooks group, no significant differences in the CECDs were found between 7 weeks postoperatively and 19 weeks postoperatively. The CECDs between preoperatively and 7 weeks postoperatively and preoperatively and 19 weeks postoperatively in the control group differed significantly. However, no significant differences in the CECDs were found between 7 and 19 weeks postoperatively in the control group.

Table 4 presents the changes in the IOP results. Between the iris hooks and control groups, no significant differences in preoperative IOP levels were found. However, the IOP levels differed significantly between the iris hooks and control groups at 7 and 19 weeks postoperatively. The preoperative and postoperative IOP levels at 7 and 19 weeks in the iris hooks and control groups differed significantly.

Table 5 shows the changes in the IOP levels of the subgroups with preoperative IOP above and below 15 mmHg in the iris hooks and control groups. The IOP levels significantly decreased at 7 and 19 weeks postoperatively in the subgroups with preoperative IOP levels below and above 15 mmHg in the iris hooks group. Moreover, the IOP levels significantly decreased at 7 and 19 weeks postoperatively in the subgroups with preoperative IOP levels below and above 15 mmHg in the control group.

No intraoperative complications or capsulorhexis tears were found in the iris hooks or control groups.

## 4. Discussion

This study demonstrated that the eight-chop technique using iris hooks had a surgical time of 10.6 min, which was shorter than the previously reported surgical times of 37 to 39 min [2]. While the use of iris hooks and the pupil expansion ring are the most time-consuming techniques in the surgery, they have the advantage of allowing a stable pupil size to be maintained throughout the procedure [7]; the pupil expansion ring is quicker to use than iris hooks [2]. In addition, the use of iris hooks and the Malyugin ring may reduce intraoperative corneal endothelial cell loss. [16]. However, Malyugin rings maintain a circular structure within the anterior chamber and have a greater potential for contact with the corneal endothelial cells compared to iris hooks. The ring thickness, in conjunction with its ring-like structure, requires careful manipulation within the eye. Pupil expansion is also limited; thus, if the lens nucleus is large and hard, it cannot be efficiently split. Iris hooks do not have a three-dimensional structure; thus, there is little possibility of contact with the corneal endothelial cells, and contact with the iris is also limited. Moreover, because the pupil diameter can be changed arbitrarily, it can be safely operated by securing the diameter appropriate for the surgeon’s needs. To effectively use iris hooks, they should be placed from the scleral side to ensure an enlarged pupil diameter range, and the iris should not be elevated (Figure 2). Therefore, apart from their insertion time, iris hooks are considered one of the best options for patients with small pupils. Phacoemulsification with the eight-chop technique takes an extremely short time, and, even with the use of iris hooks, surgical involvement may be very low.

The present study also measured the iris hooking time, which was 3.3 min. The eight-chop technique using iris hooks had a shorter phaco time and a lower cumulative dissipated energy, and it used only one-third to one-sixth of the volume of fluid that was used with the other techniques [3,17,18]. In particular, a smaller volume of fluid used may result in less surgical involvement of the cells of the trabecular meshwork and Schlemm’s canal, including corneal endothelial cells, due to the shorter time required to enter the ultrasound and irrigation/aspiration tip into the eye.

Because it represents the true summation of intraocular insult during surgery, CECD assessment is critical for comparing different techniques [13,19]. A 5% to 16% reduction in CECD was reported after cataract surgery in the first months after surgery [3,13,18,20,21]. In this study, the reduction was only 2.6% and 2.1%, respectively, at 7 and 19 weeks after surgery in the iris hooks groups and 1.3% and 1.2%, respectively, at 7 and 19 weeks after surgery in the control groups. However, significant differences were found in the decrease in CECD postoperatively between the iris hooks and control groups. This may be due to the iris hook contacting intraocular tissue, increasing postoperative inflammation and reducing CECD, because the difference in fluid volume used was only 10 mL. Mechanical stimulation of the iris during cataract surgery may indirectly affect the corneal endothelial cells.

After phacoemulsification cataract extraction and intraocular lens implantation in patients with cataract, many investigators have reported a lowering of IOP [22,23]. IOP reductions of 4–10% have been demonstrated [15,24,25]. Postoperative IOP changes are proportional to preoperative IOP. However, Poley et al. [15,26] reported an increase in the IOP in the primary open-angle glaucoma and normal groups at 1 year compared to that of the preoperative levels. The IOP-lowering rates at 19 weeks postoperatively in the present study were 12.0% and 18.1% in the iris hooks and control groups, respectively, which were higher than previously reported data in both groups. In addition, the iris hooks and control groups with an IOP of less than 15 mmHg had a significant decrease in IOP at 7 and 19 weeks after surgery. This higher IOP reduction may have been due to the eight-chop technique that minimizes ocular surgical involvement of the intraocular tissues, including the trabecular meshwork and Schlemm’s canal, compared to other techniques. Phacoemulsification has the potential to lower postoperative IOP; the higher the preoperative IOP, the greater the IOP-lowering effect of phacoemulsification. However, surgical intervention may have an impact on the IOP-lowering effect of phacoemulsification. Poley et al. were unable to detect IOP reduction in the group with the lowest preoperative IOP, indicating that the surgical involvement of the technique used may have significantly confounded the effect of IOP reduction. The eight-chop technique with or without iris hooks is less invasive and may provide the IOP-lowering effect of phacoemulsification. Significant differences were found in the decrease in the IOP postoperatively between the iris hooks and control groups. The 6.1% difference in the IOP reduction between the iris hooks and control groups suggests that the use of iris hooks may reduce the postoperative IOP decrease by approximately 6% due to the effect on intraocular tissue. Mechanical stimulation of the iris during cataract surgery could indirectly affect the trabecular meshwork cells.

The phacoemulsification techniques employed in previous reports in patients with small pupils are the divide-and-conquer [27,28], phaco-chop [7,29], step-by-step chop [3], stop-and-chop [30], quick-chop [30], or unknown [2,5,15,16,31,32,33,34,35] techniques. The prechop and eight-chop techniques have not been used in any studies to date. Phaco time, aspiration time, cumulative dissipated energy, and volume of fluid used, including operative time, should be recorded when studying changes in IOP and CECD after phacoemulsification cataract surgery. However, to my knowledge, intraoperative parameters in patients with small pupils have not been reported in previous studies on the effects of phacoemulsification cataract surgery on the IOP and CECD. Therefore, it is imperative to investigate the alterations in IOP and CECD subsequent to phacoemulsification cataract surgery when a highly sophisticated phacoemulsification technique is utilized, which aims to minimize the surgical involvement of intraocular tissues.

The presence of small pupils is associated with an increased risk of complications, including posterior capsule rupture and vitreous loss [5,36,37]. Balal et al. [5] reported that among 20,175 patients with cataracts, 6.7% and 3.8% of those in whom iris hooks and the Malyugin ring were used, respectively, experienced posterior capsule rupture. Thus, posterior capsule rupture occurred in 4.7% of all the patients in whom iris hooks or the Malyugin ring were used [5]. However, none of the 65 patients in the current study had any complications, including posterior capsule rupture. Therefore, the eight-chop technique using iris hooks could be an excellent method concerning safety, in addition to reducing surgical involvement and lowering IOP.

Results of this study have not been compared to studies using the divide-and-conquer or phaco-chop techniques or to studies using other mechanical pupil dilation techniques. This must be taken into account when interpreting the current results. Nevertheless, numerous additional studies have employed the divide-and-conquer and phaco-chop techniques in conjunction with alternative mechanical pupil dilation techniques.

In the current study, the pupil diameter of patients averaged 5.34 mm, which was larger than that previously reported [7,16]. However, the average pupil diameter of the control group was 7.62 mm; thus, it was reduced by 25.1% for the iris hooks group. Therefore, this pupil diameter could be considered challenging for surgical management. Many cases of small pupils encountered in clinical practice are caused by diabetes mellitus, pseudoexfoliation syndrome, or intraoperative floppy iris syndrome [29]. Hence, the patients with small pupils in the present study were considered appropriate for assessing the eight-chop technique. Furthermore, if the greatest priority is surgical safety, mechanical pupil expansion, such as using iris hooks and Malyugin rings, should be considered for patients with a pupil size of <6 mm.

## 5. Conclusions

The eight-chop technique allows for efficient phacoemulsification while protecting the corneal endothelial cells with an ophthalmic viscosurgical device, keeping the anterior chamber stable, restricting the movement of the lens nucleus and breaking it into smaller pieces to reduce the total ultrasound energy. In this study, intraoperative outcome measures were excellent, the reduction in CECD was small, and the postoperative IOP reductions were maintained. It may therefore be concluded that the eight-chop technique employing iris hooks represents an optimal solution for patients with small pupils.

## Figures and Tables

**Table 1:** Preoperative characteristics and intraoperative parameters.

Characteristic/Parameter	Iris Hooks Group	Control Group	*p*-Value
Number of eyes	65	65	
Age (y)	76.8 ± 7.8	75.0 ± 6.2	0.16 ^a^
Gender: Men	42 (65%)	16 (25%)	<0.01 ^b^
Women	23 (35%)	49 (75%)	
Diabetes mellitus	15	10	0.27 ^c^
IFIS	10	0	<0.01 ^b^
Preoperative pupil size (mm)	5.34 ± 0.53	7.62 ± 0.55	<0.01 ^d^
Postoperative pupil size (mm)	6.37 ± 0.68	7.33 ± 0.65	<0.01 ^d^
Iris hooking time (min)	3.3 ± 1.37	-	
Operative time (min)	10.6 ± 2.32	4.6 ± 1.20	<0.01 ^d^
Phaco time (s)	20.7 ± 6.4	16.2 ± 7.6	<0.01 ^d^
Aspiration time (s)	101.1 ± 52.2	72.1 ± 18.6	<0.01 ^d^
CDE	7.8 ± 2.44	7.0 ± 3.36	0.16 ^a^
Volume of fluid used (mL)	38.0 ± 8.8	28.9 ± 9.2	<0.01 ^d^

Unless otherwise noted, values are expressed as mean ± standard deviation or as percentages. ^a^ Unpaired *t*-test showed no significant differences between the groups. ^b^ Chi-square test showed significant differences between the groups. ^c^ Chi-square test showed no significant differences between the groups. ^d^ Unpaired *t*-test showed significant differences between the groups. IFIS, intraoperative floppy iris syndrome; CDE, cumulative dissipated energy.

**Table 2:** Pre- and postoperative best-corrected visual acuity values.

Group	Preoperatively	7 Weeks Postoperatively	19 Weeks Postoperatively	*p-*Value
Iris hooks (logMAR) (*n* = 65)	0.21 ± 0.33	−0.011 ± 0.083	−0.026 ± 0.078	<0.01 ^a^, <0.01 ^a^, <0.01 ^a^
Control (logMAR) (*n* = 65)	0.17 ± 0.29	−0.042 ± 0.074	−0.039 ± 0.075	<0.01 ^a^, 0.42 ^b^, <0.01 ^a^
*p*-Value	0.47 ^c^	<0.05 ^d^	0.38 ^c^	

Values represent mean ± standard deviation. *p*-Values in the right column are presented in the same order: preoperatively vs. 7 weeks postoperatively, 7 weeks postoperatively vs. 19 weeks postoperatively, and preoperatively vs. 19 weeks postoperatively. ^a^ Paired *t*-test showed significant differences between the groups. ^b^ Paired *t*-test showed no significant differences between the groups. ^c^ Unpaired *t*-test showed no significant differences between the groups. ^d^ Unpaired *t*-test showed significant differences between the groups. logMAR, logarithmic minimum angle of resolution.

**Table 3:** Pre- and postoperative corneal endothelial cell density values.

Group	Preoperatively	7 Weeks Postoperatively	19 Weeks Postoperatively	*p-*Value
Iris hooks (cells/mm^2^) (*n* = 37)	2503.6 ± 213.0	2437.4 ± 215.6	2448.8 ± 215.7	<0.01 ^a^, 0.51 ^b^, <0.01 ^a^
loss (%)	-	2.6 ± 4.3	2.1 ± 4.4	-
Control (cells/mm^2^) (*n* = 65)	2585.1 ± 236.2	2553.2 ± 254.6	2553.2 ± 246.0	<0.01 ^a^, 0.99 ^b^, <0.01 ^a^
loss (%)	-	1.3 ± 3.2	1.2 ± 3.2	-
*p*-Value	0.09 ^c^	<0.05 ^d^	<0.05 ^d^	

Values represent mean ± standard deviation. *p*-Values in the right column are presented in the same order: preoperatively vs. 7 weeks postoperatively (upper left), 7 weeks postoperatively vs. 19 weeks postoperatively (upper right), and preoperatively vs. 19 weeks postoperatively (lower). ^a^ Paired *t*-test showed significant differences between the groups. ^b^ Paired *t*-test showed no significant differences between the groups. ^c^ Unpaired *t*-test showed no significant differences between the groups. ^d^ Unpaired *t*-test showed significant differences between the groups.

**Table 4:** Mean intraocular pressure and mean reduction in intraocular pressure in the course of time.

	Mean IOP (mmHg) ± SD (% Decrease)						
Examination	Iris Hooks Group (*n* = 51)		Control Group (*n* = 65)		*p*-Value		
Preoperatively	14.5 ± 2.5	-	14.0 ± 2.2	-	0.33 ^a^		
7 weeks postoperatively	13.0 ± 2.7	(10.1 ± 15.4)	11.8 ± 2.3	(15.6 ± 12.3)	<0.01 ^b^	<0.01 ^c^	<0.01 ^d^
19 weeks postoperatively	12.7 ± 2.6	(12.0 ± 11.8)	11.5 ± 2.4	(18.1 ± 9.7)	<0.01 ^b^	<0.01 ^c^	<0.01 ^d^

^a^ Unpaired *t*-test showed no significant differences between the groups. ^b^ Unpaired *t*-test showed significant differences between the groups. ^c^ Paired *t*-test showed significant differences between the preoperative and respective time values in the iris hooks group. ^d^ Paired *t*-test showed significant differences between the preoperative and respective time values in the control group. IOP, intraocular pressure; SD, standard deviation.

**Table 5:** Mean intraocular pressure and mean decrease in the groups with preoperative intraocular pressure levels above and below 15 mmHg of the iris hooks and control groups.

	Iris Hooks Group					
Examination	IOP Above 15 mmHg Group (mmHg) (*n* = 26)		*p-*Value	IOP Below 15 mmHg Group (mmHg) (*n* = 25)		*p*-Value
Preoperatively	16.5 ± 1.4	(%)		12.5 ± 1.6	(%)	
7 weeks postoperatively	14.6 ± 2.0	(11.3 ± 10.8)	<0.01 ^a^	11.3 ± 2.4	(8.9 ± 19.1)	<0.01 ^a^
19 weeks postoperatively	14.4 ± 1.9	(12.3 ± 11.2)	<0.01 ^a^	11.0 ± 2.0	(11.6 ± 12.7)	<0.01 ^a^
	**Control Group**					
**Examination**	**IOP above 15 mmHg Group** **(mmHg)** **(*n* = 27)**		***p-*** **Value**	**IOP Below 15 mmHg Group** **(mmHg)** **(*n* = 38)**		***p*** **-Value**
Preoperatively	16.0 ± 0.9	(%)		12.7 ± 1.3	(%)	
7 weeks postoperatively	12.8 ± 2.0	(20.4 ± 12.1)	<0.01 ^a^	10.8 ± 1.9	(14.9 ± 13.0)	<0.01 ^a^
19 weeks postoperatively	12.8 ± 1.8	(20.2 ± 10.7)	<0.01 ^a^	10.6 ± 1.8	(16.7 ± 9.6)	<0.01 ^a^

Unless otherwise noted, values are expressed as mean ± standard deviation or as percentages. ^a^ Paired *t*-test showed significant differences between the preoperative and respective time values. IOP, intraocular pressure.

## Data Availability

The data presented in this study are available on request from the corresponding author due to privacy and ethical restrictions.
